# Telemedicine Adoption, US Ambulatory Visits, and Total Medical Spending, 2019-2023

**DOI:** 10.1001/jamanetworkopen.2026.11835

**Published:** 2026-05-11

**Authors:** John N. Mafi, Sitaram Vangala, Jonathan Cantor, Melody Craff, Manying Cui, Artem Romanov, Ziyi Li, Michelle Rockwell, Chi-Hong Tseng, Dale Skinner, Michael Hadfield, A. Mark Fendrick, Cheryl L. Damberg, Catherine Sarkisian, Katherine L. Kahn

**Affiliations:** 1 Division of General Internal Medicine and Health Services Research, David Geffen School of Medicine at UCLA, Los Angeles, California; 2 RAND, Santa Monica, California; 3 MedInsight, Milliman Inc, Seattle, Washington; 4 Family & Community Medicine, Virginia Tech Carilion School of Medicine, Roanoke; 5 Internal Medicine, Health Management and Policy, University of Michigan Medical School, Ann Arbor

## Abstract

**Importance:**

Telemedicine is now widely used, stimulated by pandemic-era expansion rules and payment parity to in-person visits. Lawmakers continue to consider how to revise existing policies because of uncertainty about the potential for telemedicine to increase utilization and spending.

**Objective:**

To quantify the association between telemedicine adoption and visits and spending.

**Design, Setting, and Participants:**

This cohort study used multipayer medical claims data from MedInsight’s research database for a national sample of adults continuously enrolled in Medicare fee-for-service, Medicare Advantage, dual-eligible, Medicaid, or commercial insurance, from January 1, 2019, to October 31, 2023. Data were analyzed from April 30, 2024, to February 10, 2026.

**Exposure:**

Regional telemedicine adoption (measured at the hospital referral region [HRR] level).

**Main Outcomes and Measures:**

The primary outcomes were (1) total combined telemedicine and in-person ambulatory visits (primary care, specialist, and preventive screening visits), and (2) total combined per-member-per-month spending on professional, inpatient, facility outpatient, prescription drug, and ancillary payments. By use of difference-in-differences analyses on visit-level and spending-level changes before (January 1, 2019, to December 31, 2019) vs after (January 1, 2021, to October 31, 2023) telemedicine expansion in high-telemedicine vs low-telemedicine adoption quintiles (measured at the HRR level), age-adjusted, sex-adjusted, and diagnosis-adjusted Poisson regressions were estimated, accounting for repeated measurements over time. Analysis was also stratified by urbanicity, payer, and Centers for Disease Control and Prevention Social Vulnerability Index quintiles to explore heterogeneous associations.

**Results:**

The sample included 3.04 million US individuals (mean [SD] age, 54.2 [17.2] years; 55.7% female) who utilized 120 million visits and incurred $178.4 billion in spending during 2019 to 2023. In 2019, the mean (SD) visit rate was 0.66 (0.035), and the mean (SD) spending rate was $774.59 ($36.78) per-member-per-month. Overall, point estimates suggested high-adopting areas had 2.4% (95% CI, −8.1% to 3.6%) fewer visits and 0.5% (95% CI, −13.1% to 13.9%) lower spending; however, 95% CIs crossed the null. Similarly, point estimates varied across subgroups but none achieved statistical significance: there were 4.4% (95% CI, −11.2% to 3.0%) fewer visits and 2.3% (95% CI, −18.9% to 17.8%) lower spending among urban populations, 2.5% (95% CI, −12.9% to 8.0%) lower spending for Medicaid-insured individuals, 5.3% (95% CI, −47.1% to 66.2%) lower spending for dual-eligible individuals, 3.0% (95% CI, −9.2% to 3.5%) lower spending for Medicare Advantage–insured individuals, and 1.5% (95% CI, −19.1% to 19.8%) lower spending among the most socially vulnerable populations. Conversely, point estimates suggested 3.4% (95% CI, −4.9% to 12.5%) greater visits and 3.8% (95% CI, −12.2% to 21.4%) higher spending in rural areas, 1.1% (95% CI, −12.8% to 17.3%) higher spending for commercially insured individuals, 1.0% (95% CI, −7.1% to 11.5%) higher spending for Medicare fee-for-service-insured individuals, and 4.5% (95% CI, −12.7% to 23.1%) higher spending among the least socially vulnerable groups. All 95% CIs crossed the null.

**Conclusions and Relevance:**

Nationwide telemedicine adoption was not significantly associated with changes in visits or spending, either overall or when stratified by urbanicity, payer type, or area-level social vulnerability, thus easing concerns about large utilization and spending increases from telemedicine expansion.

## Key Points

**Question:**

Is telemedicine adoption associated with health care visits and spending, and does the association vary across different insured populations?

**Findings:**

In this cohort study using difference-in-differences analyses of over 3 million US adults during 2019 to 2023, point estimates suggested that high-telemedicine-adopting areas had 2.4% fewer visits and 0.5% lower spending; however, the 95% CIs crossed the null. Utilization and spending changes were consistently null across Medicare fee-for-service, Medicare Advantage, Medicaid, dually eligible, and commercially insured populations.

**Meaning:**

These findings suggest that telemedicine adoption is not significantly associated with changes in visits or spending, either overall or across major payer groups, easing concerns about large spending increases.

## Teaser

This cohort study examines the association between telemedicine adoption and health care visits and spending.

## Introduction

The declaration of the COVID-19 pandemic national Public Health Emergency (PHE) in 2020 triggered the rapid proliferation of telemedicine. Key federal policy changes contributed to this growth, specifically the telemedicine flexibilities of the Centers for Medicare & Medicaid Services (CMS), including payment parity for telemedicine and in-person visits, waiving geographic restrictions, and eliminating patient out-of-pocket cost-sharing.^1,2,3,4,5,6,7,8,9,10^ The expiration of the PHE on May 11, 2023, however, introduced uncertainty about telemedicine’s future, and policymakers implemented several temporary extensions of these flexibilities in order to gather information about telemedicine’s impact on health care utilization and spending. Absent congressional action, the CMS telemedicine flexibilities will expire in 2027.^11,12^

As lawmakers debate whether to permanently extend or modify the CMS telemedicine flexibilities for the millions of US individuals who use telemedicine for their care,^13^ they will weigh several questions, specifically how telemedicine affects ambulatory care utilization and total medical spending.^14,15,16,17^ By making care more convenient, telemedicine could increase utilization—and therefore spending—for self-limiting conditions^18^; however, it may reduce spending by shifting patients from high-cost emergency department or office visits to brief, lower-intensity virtual evaluations.

Moreover, several studies^6,19,20,21,22,23,24,25,26^ raise concerns that telemedicine could exacerbate health care disparities.^15,18,21^ Previous studies have found differences in telemedicine use by county urbanicity^27^ and at the state level because of payment parity mandates.^28^ Our prior study^2^ identified both rapid telemedicine adoption and deepening disparities in US care access (from January 1, 2018, through February 28, 2021), raising the question of telemedicine adoption’s heterogeneous impact across a wide sample of US individuals.^29^

Recognizing that federal policy decisions about CMS’s continued payment for telemedicine will reverberate across the entire US health care system,^13^ understanding telemedicine’s association with visits and spending across both public and private payers will inform the national debate on telemedicine policymaking. Building on our prior work,^2^ and using analytic approaches similar to those of previous studies,^16,17^ we present the first, to our knowledge, holistic examination of telemedicine’s association with nationwide visits and spending across 5 major payer classes throughout the entire PHE. We also determine whether the association varied by payer and demographic patient and county-level subgroups. We hypothesized that telemedicine adoption would be associated with heterogeneous effects on visits and spending, with increased visits and spending among those typically enrolled in fee-for-service (FFS) insurance plans, and reduced visits and spending among groups commonly enrolled in capitated managed care plans.

## Methods

In this cohort study, we analyzed multipayer medical claims data from the Milliman MedInsight Emerging Experience Research Database (hereafter, the MedInsight research database) across all 50 US states from January 1, 2019, to October 31, 2023.^2^ The UCLA institutional review board deemed this analysis exempt from review and the need for informed consent. This study follows the Strengthening the Reporting of Observational Studies in Epidemiology (STROBE) reporting guidelines for cohort studies.

### Data Source and Cohort

The MedInsight research database represents a large national convenience sample of 75 participating health care organizations that purchase MedInsight data analysis services and allow use of monthly deidentified claims data for research purposes (eFigure 1 in Supplement 1). Demographics of the database were shown to be largely comparable to data from the American Community Survey (eFigure 2 in Supplement 1), and the MedInsight research database has previously been analyzed to study US health care utilization and spending patterns during the PHE.^2,30,31,32,33^

The primary analysis cohort included adult patients aged 18 years and older with continuous coverage in Medicare FFS, Medicare Advantage (MA), Medicare-Medicaid (dual-eligible), Medicaid, or commercial insurance between January 1, 2018, to October 31, 2023, in all 50 US states (2018 data enabled pre–parallel trends testing). Dual-eligible beneficiaries were enrolled in either Medicare FFS or MA. The longitudinal study design enhances internal validity by observing an unchanging cohort of patients over time, allowing more precise and quasi-experimental estimates of telemedicine adoption’s association with ambulatory visits and total medical spending, before (2019) vs after (2021-2023) telemedicine expansion.

Missing patient or zip code level sociodemographic data was generally low: 0.76% of patients had missing sex data and 3.9% had missing Social Vulnerability Index (SVI) data. The eMethods in Supplement 1 contain further details on missing data.

### Exposure

During March 2020, 2 major events occurred simultaneously: the onset of COVID-19 and telemedicine expansion. To evaluate the association of telemedicine adoption with visits and spending, we followed an approach similar to that used in previous telemedicine research.^17,34,35^ Building on prior work leveraging regional-level, health system–level and practice-level telemedicine adoption,^17,34,36,37^ patients were assigned to Dartmouth Health Atlas^38^ hospital referral regions (HRRs) on the basis of their 2024 home address (as older information was not available), and exposure was defined as a patient’s HRR belonging to the top quintile of 2020 telemedicine adoption. The 306 HRRs are widely used in health services research to reflect tertiary health care delivery systems, and are generally regarded as a more precise indicator of health care markets than states or counties.^36,39,40,41,42^ HRRs were cross-walked to patients’ 5-digit zip codes by Milliman MedInsight and then were rolled up into telemedicine adoption quintiles. Each patient’s 5-digit zip code was mapped to a specific HRR via the Dartmouth crosswalk, and each HRR was then classified into a telemedicine adoption quintile on the basis of its 2020 telemedicine use rate. We examined whether changes in patients’ addresses during the PHE could influence our results, and we found that moving rates were reassuringly low, with 5.4% of patients switching zip codes (ie, moving) during January 2, 2021, to December 31, 2023. Our definition of telemedicine use included video, audio-only, and electronic visits of any type, including office visits, urgent care, behavioral health, among other types (see eMethods in Supplement 1).

The group of patients living in the quintile with the highest telemedicine use in 2020 (telemedicine adoption’s peak year) served as the exposure group for exposure to both the effects of COVID-19 and telemedicine expansion, consistent with prior work related to telemedicine and the PHE.^17,34,35^ We classified as the comparison group patients living in the quintile with the lowest telemedicine adoption in 2020, regardless of their pre-PHE visit history.

### Measures

We measured total ambulatory visits (visits) as a rate, defined as the sum of in-person plus telemedicine utilization including primary care, specialist, and US Preventive Services Task Force Grade A/B-recommended and Health Resources and Services Administration–recommended preventive screening examination visits per member per month (PMPM). Each service receives an equal weight (of 1).^43^ Emergency department visits were analyzed separately (eMethods in Supplement 1). The eMethods and eTable 1 in Supplement 1 provide measure validation details.

We also assessed total medical spending (spending) by calculating the total paid amount to physicians and hospitals (including patients’ out-of-pocket costs) summed across payers. Spending was expressed as expenditures PMPM, combining professional (eg, clinician-associated services), inpatient (eg, hospitalizations), facility outpatient (eg, outpatient hospital department services), prescription drug (eg, antihypertensive drugs), and ancillary (eg, home health services) expenditures PMPM. We normalized spending to 2023 inflation rates using the US Bureau of Labor Statistics Consumer Price Index calculator.^44^

### Statistical Analysis

#### Overview

We implemented a difference-in-differences design,^45^ consistent with our and others’ prior work on telemedicine during the PHE.^2,17,34,35^ We contrast relative changes in monthly visit and PMPM spending for patients living in the highest quintile of telemedicine adoption (exposure group) and those living in the lowest quintile of telemedicine adoption (comparison group) before (2019) vs after (2021-2023) telemedicine expansion. The comparison group served as the counterfactual for experiencing the PHE’s effects with relatively low telemedicine exposure. Importantly, our analysis of relative (rather than absolute) changes in visit and spending rates accounted for preexisting differences in baseline levels between those living in the highest vs lowest telemedicine-using quintiles.

#### Multivariable Adjustment

We used age-adjusted and sex-adjusted Poisson regression models with offsets for total patient-months to estimate a ratio of rate ratios (RoRR). An RoRR of 1 means that no change in either visits or spending from before to after pandemic onset has occurred. RoRRs less than 1 signify declines in the dependent variable (eg, visit rates or spending) and may be interpreted as a percentage decline. For example, an RoRR of 0.96 is interpreted as a 4% decrease vs prepandemic rates. Conversely, RoRRs greater than 1 signify increases in the dependent variable. An RoRR of 1.11 would be interpreted as an 11% increase. We implemented cluster-robust SEs to account for repeated measurements over time. Because our data are structured at the telemedicine adoption quintile by patient characteristics by time level, we clustered at the telemedicine adoption quintile by patient characteristics level, so that repeated measures of each aggregated group of patients over time are properly accounted for. Although the underlying sample comprises millions of unique individuals, the unit of observation in our regressions is the aggregated cell defined by telemedicine adoption quintile, patient characteristics, and time period, with each cell including multiple HRRs within a quintile. This approach also provides model-robust inferences, addressing potential misspecification of the Poisson error model for spending outcomes. To evaluate the overall association of telemedicine adoption with visits and spending, averaged over the effects within urban and rural subgroups, we also adjusted for an urbanicity main effect in addition to using an urbanicity and time interaction effect. Importantly, we also identified and adjusted for potential differences in population health by including baseline medical diagnoses that were present before the PHE, from January 1, 2018, to February 29, 2020. We identified these using *International Statistical Classification of Diseases and Related Health Problems, Tenth Revision,* diagnosis codes categorized by the Agency for Healthcare Research and Quality Clinical Classifications Software Refined list of body systems.^46^

**Figure 1.**
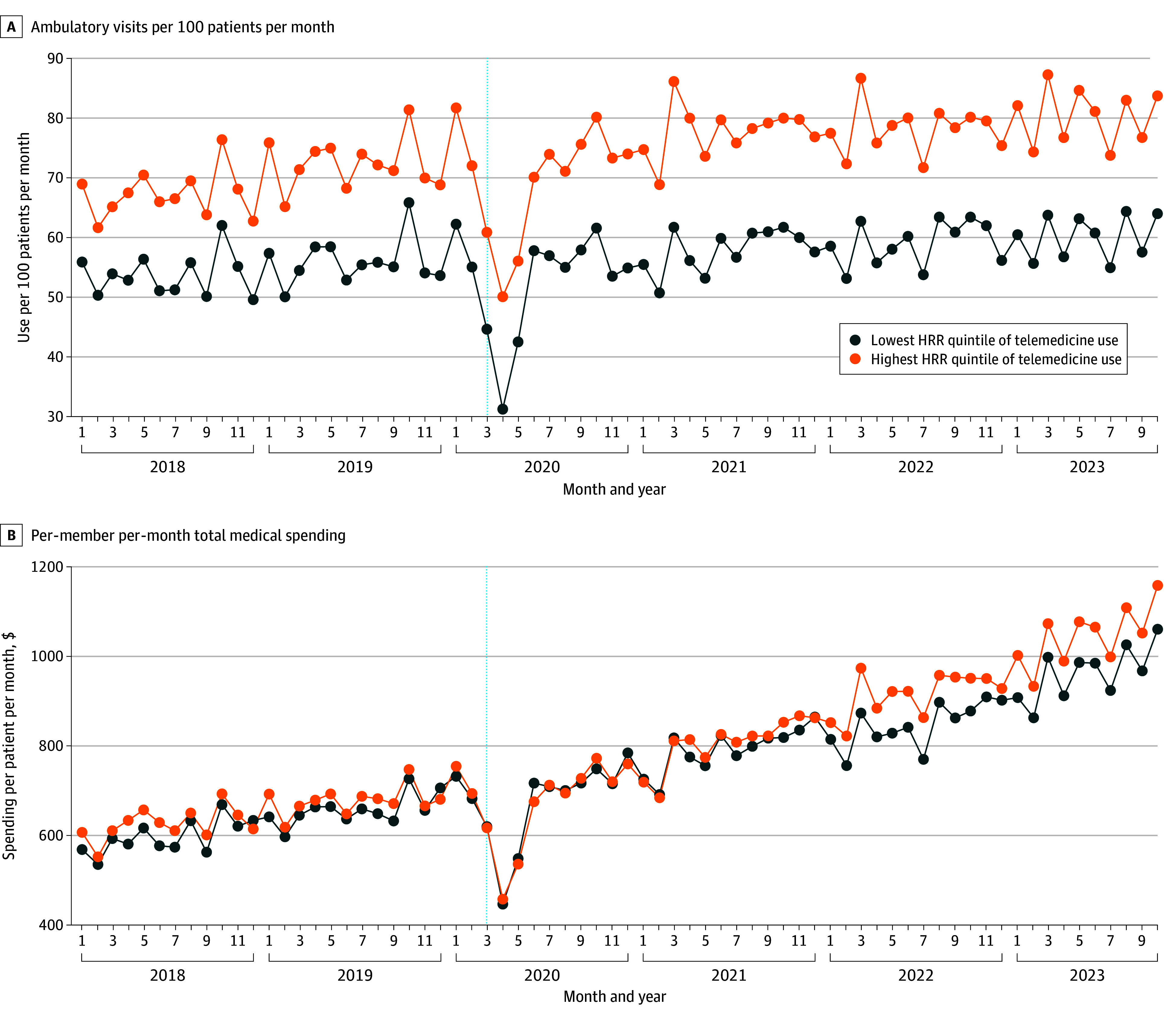
Line Graphs of Crude Ambulatory Visits and Total Spending Figure shows ambulatory visits (A) and total spending rates (B) per 100 patients per month living in the hospital referral zones (HRRs) with the highest vs the lowest telemedicine adoption quintiles in 2020 for all 3.04 million patients from January 1, 2018, through October 31, 2023. See the Methods for an explanation of how HRRs are determined. The vertical line indicates March 11, 2020, which is when the World Health Organization declared the onset of the COVID-19 pandemic. These tests yielded visually pre–parallel trends and quantitatively approximately parallel prepandemic trends, with a nonsignificant 1.0% (95% CI, −3.4% to 3.2%) decline in visits and a 1.7% (95% CI, −8.5% to 13.1%) increase in spending in 2018 vs 2019 among those living in the highest vs lowest telemedicine-using quintiles (eTables 6-9 in Supplement 1). Spending is normalized to 2023 dollars using the Consumer Price Index Inflation Calculator of the US Bureau of Labor Statistics.^44^

These multivariable-adjusted RoRRs describe telemedicine adoption-associated changes in visit and spending rates. RoRRs provide an estimate of observed-vs-expected visit or spending rates based on the assumption that, absent telemedicine expansion, visit or spending trends in the highest quintile of telemedicine use, would have parallel trends to the lowest quintile of 2020 telemedicine use. To test this assumption, we compared pre-PHE visit and spending rates in 2018 vs 2019. These placebo tests yielded visually parallel pre-PHE trends^17^ (Figure 1; eFigure 3 in Supplement 1) and quantitatively approximately parallel pre-PHE trends (eTables 2-5 in Supplement 1).

Pre-PHE trends were generally parallel among all study subgroups except for behavioral health and substance use disorder visits and spending, which were therefore both excluded as outcomes in our main analyses. Still, we included the behavioral health and substance use disorder visits when defining regional telemedicine adoption quintiles and in our spending analyses.

#### Comparing Utilization and Spending Associations by Socioeconomic Subgroups

To assess the heterogeneous impacts of telehealth expansion, we stratified the estimates of changes in visits and spending by patient geography (eg, urbanicity and US census region), payer, and social vulnerability.^47,48,49^ We measured urbanicity using the 2023 Rural-Urban Continuum Codes, and cross-walked 5-digit zip codes to Rural-Urban Continuum Codes, defining codes 1 to 3 as urban and codes 4 to 10 as rural. Furthermore, we defined social vulnerability using the Centers for Disease Control and Prevention’s SVI quintiles at the zip code level.^48^ We compared telemedicine’s associations among those living in the most vs least socially vulnerable quintiles (the eMethods in Supplement 1 provide details on cross-walking zip codes). Conservatively, differences were considered statistically significant when the 95% CIs did not overlap between subgroups.

To mitigate time-varying confounding, we performed sensitivity analyses redefining the regional telemedicine exposure variable by quantifying telemedicine exposure in 2022 (January 1, 2022, to December 31, 2022), while still comparing 2019 vs 2023 consistent with prior work.^35^ We anticipated that there would be less time-varying confounding during 2022 than during 2020. If results from these models remained consistent with the main findings, the results would provide additional reassurance against the possibility of time-varying confounding. Additionally, we conducted a negative control analysis examining injury-related spending (INJ category in the Clinical Classifications Software Refined classification). Injury and trauma care has no plausible telehealth substitution pathway, as fractures, lacerations, and other trauma require in-person care. If our identification strategy were valid, we would expect no differential changes in injury spending between high and low telehealth adoption regions.

To address the problem of multiple comparisons, we applied the Benjamini-Hochberg step-up procedure to control the false-discovery rate in each set of stratified analyses at the 5% level.^2,50^ All statistical analyses were performed at UCLA using R statistical software version 3.6.2 (R Project for Statistical Computing) between April 30, 2024, and February 10, 2026.

## Results

We examined data on 3.04 million insured US adults (mean [SD] age, 54.2 [17.2] years; 55.7% female) who utilized 120 million visits (9.8% were telemedicine) and incurred $178.44 billion in spending between January 1, 2019, to October 31, 2023. Mean (SD) pre-PHE (2019) PMPM visit rates were 0.66 (0.035), and mean (SD) spending rates were $774.59 ($36.78) (eFigure 4 in Supplement 1). In total, 84.3% of patients were urban, 14.0% were rural, and 1.8% had unknown urbanicity. In 2020, telemedicine comprised 40.7% of total ambulatory visits in the highest quintile of telemedicine adoption (vs 0.26% of visits in 2019) and 2.5% of visits in the lowest quintile of telemedicine adoption (vs 0.24% of total ambulatory visits in 2019) (Table 1 and Figure 1).

**Table 1.** Unadjusted Demographic Characteristics Stratified by Quintiles of 2020 Telemedicine Use^a^

Category, strata	Patients, % (N = 3.04 million)					
	Quintile 1 (low)	Quintile 2	Quintile 3	Quintile 4	Quintile 5 (high)	Unknown
Age group, y						
18-39	18.3	21.2	22.5	24.2	23.8	17.7
40-64	40.1	42.4	47.2	46.1	47.2	38.3
65-79	32.5	29.2	24.0	23.3	22.2	31.6
≥80	9.0	7.2	6.3	6.3	6.8	12.4
Gender						
Female	54.9	54.6	55.5	56.1	56.1	40.1
Male	45.1	45.3	44.3	43.4	43.4	32.5
Unknown	0.0	0.0	0.2	0.5	0.5	27.4
SVI vulnerability percentile						
81-100	5.8	8.9	8.9	12.7	10.1	0.0
61-80	15.1	13.5	16.0	16.0	13.6	0.0
41-60	31.6	17.5	19.5	17.0	15.8	0.0
21-40	27.2	27.3	27.4	20.9	24.9	0.0
1-20	1.3	2.5	2.1	1.5	2.8	0.0
Unknown overall SVI	1.7	15.1	12.1	8.3	19.4	100.0
Coverage						
Commercial	52.2	54.7	57.1	50.0	57.5	36.2
Medicare-Medicaid	1.1	2.0	3.9	3.6	2.5	14.3
Medicaid	6.2	8.4	11.6	20.8	14.7	15.7
Medicare Advantage	29.0	18.3	18.3	17.9	9.7	31.8
Medicare fee for service	11.5	16.7	9.2	7.7	15.5	2.1
Urbanicity						
Rural	52.6	20.0	14.7	9.3	5.4	0.3
Urban	47.4	80.0	85.3	90.7	94.2	0.2
Unknown	0.0	0.0	0.0	0.0	0.4	99.5
Census region						
Midwest	60.5	73.6	41.8	42.3	12.6	0.0
Northeast	5.6	11.1	9.2	6.8	53.2	0.0
South	28.9	15.1	37.5	19.9	6.1	0.0
West	5.0	0.2	11.5	31.1	28.1	0.0
Unknown	0.0	0.0	0.0	0.0	0.0	100.0
No. of medical diagnoses^b^						
0	2.2	2.2	2.4	2.2	2.4	1.7
1	6.0	5.4	5.7	5.5	5.7	4.7
2	6.4	6.1	6.4	6.3	6.5	5.0
3	7.0	7.0	7.4	7.2	7.4	5.6
4	7.7	7.7	8.1	7.9	8.0	6.6
≥5	64.8	65.7	63.5	63.1	62.5	64.1
Unknown	6.0	5.9	6.5	7.7	7.6	12.3

Abbreviation: SVI, Social Vulnerability Index.

^a^ Although the cohort was continuously insured from 2018 to 2023, the main analyses compared visit and spending data before (2019) vs after (2021-2023) telemedicine expansion among those living in hospital referral regions with high vs low telemedicine use in 2020. eTable 10 in Supplement 1 provides counts of subgroups.

^b^ No. of diagnoses was determined using the Agency for Healthcare Research and Quality Clinical Classifications Software Refined Body System Diagnosis Count.^46^

**Figure 2.**
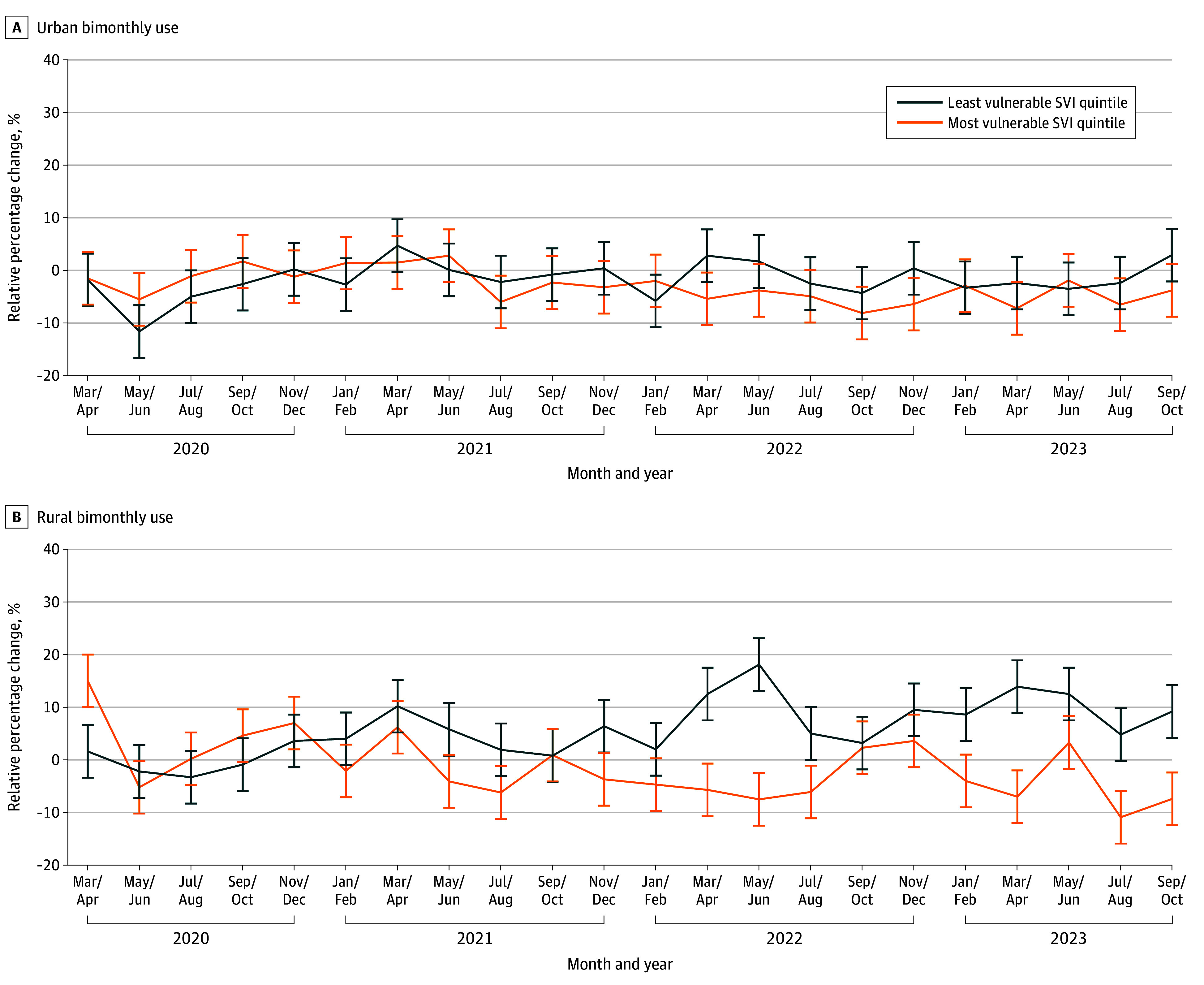
Line Graphs of Telemedicine’s Association With Ambulatory Visits in Rural and Urban Settings, Stratified by Social Vulnerability Index (SVI) Quintiles, 2019-2023 Error bars denote 95% CIs.

**Figure 3.**
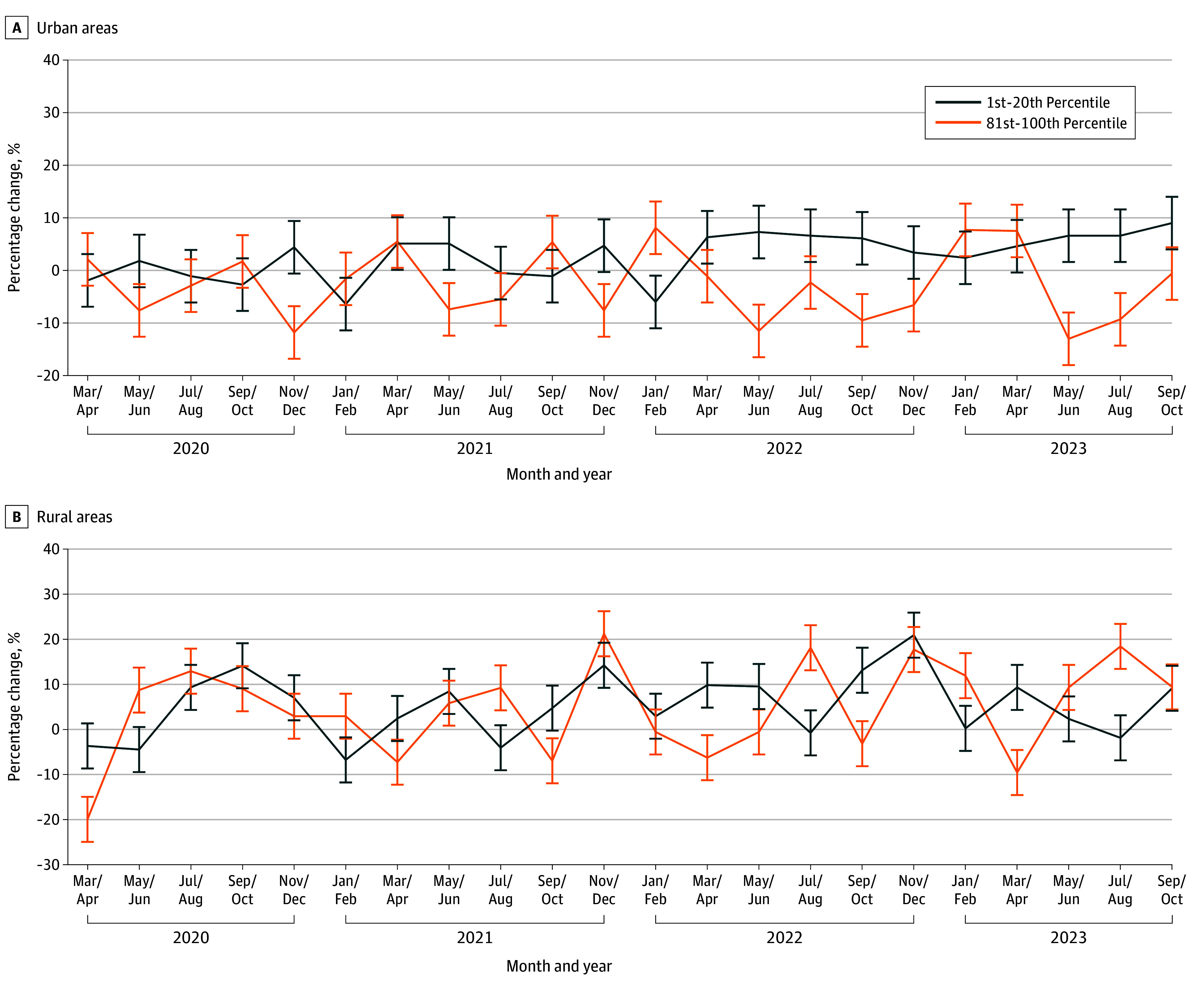
Line Graphs of Telemedicine’s Association With Total Medical Spending in Rural and Urban Settings, Stratified by Social Vulnerability Index (SVI) Quintiles, 2019-2023 Error bars denote 95% CIs.

Overall, point estimates suggested that high-adopting areas had 2.4% (95% CI, −8.1% to 3.6%) fewer visits and 0.5% (95% CI, −13.1% to 13.9%) lower spending; however, the 95% CIs crossed the null. Point estimates varied by geography, insurance types, and SVI; however, the 95% CIs consistently crossed the null (Table 2, Figure 2, and Figure 3).

**Table 2.** Association of Telemedicine With Ambulatory Visits and Total Medical Spending by Urbanicity and Stratified by SVI Quintiles, Payer, Visit Type, and Spending Type, 2019-2023 (N = 3.04 million)

Variable	RoRR (95% CI)^a^		
	Overall	Urban (84.3%)	Rural (14.0%)
Ambulatory visits			
All patients	0.98 (0.92-1.04)	0.96 (0.89-1.03)	1.03 (0.95-1.13)
SVI quintile percentile			
81-100 (Most vulnerable)	0.97 (0.90-1.04)	0.96 (0.89-1.05)	0.97 (0.86-1.09)
61-80	0.91 (0.85-0.98)	0.90 (0.82-0.98)	0.96 (0.88-1.05)
41-60	0.99 (0.93-1.04)	0.96 (0.89-1.03)	1.07 (0.98-1.16)
21-40	0.98 (0.91-1.05)	0.96 (0.89-1.04)	1.03 (0.95-1.13)
1-20 (Least vulnerable)	1.01 (0.95-1.08)	0.99 (0.92-1.07)	1.08 (0.99-1.18)
Payer			
Commercial	0.96 (0.87-1.06)	0.93 (0.81-1.07)	1.04 (0.92-1.16)
Dual	0.95 (0.72-1.25)	0.94 (0.67-1.33)	1.01 (0.82-1.25)
Medicaid	0.98 (0.92-1.05)	1.01 (0.93-1.09)	0.93 (0.85-1.03)
Medicare Advantage	0.96 (0.90-1.03)	0.92 (0.86-0.99)	1.09 (1.03-1.16)
Medicare fee-for-service	1.01 (0.97-1.05)	1.00 (0.95-1.04)	1.05 (0.97-1.14)
Visit type			
Primary care office visit	0.95 (0.86-1.06)	0.93 (0.82-1.05)	1.05 (0.96-1.14)
Specialist office visit	1.02 (0.90-1.16)	1.01 (0.87-1.18)	1.05 (0.94-1.17)
Emergency department visit (measured separately from overall visits)	0.89 (0.77-1.03)	0.90 (0.77-1.04)	0.88 (0.74-1.04)
Preventive screening	0.97 (0.87-1.08)	0.94 (0.83-1.07)	1.03 (0.89-1.20)
Total medical spending			
All patients	0.99 (0.87-1.14)	0.98 (0.81-1.18)	1.04 (0.89-1.21)
SVI quintile percentile			
81-100 (Most vulnerable)	0.99 (0.81-1.20)	0.96 (0.81-1.20)	1.06 (0.87-1.28)
61-80	0.89 (0.75-1.05)	0.85 (0.75-1.05)	0.99 (0.81-1.20)
41-60	0.98 (0.88-1.10)	0.96 (0.88-1.10)	1.04 (0.88-1.24)
21-40	1.03 (0.89-1.18)	1.03 (0.89-1.18)	1.03 (0.87-1.22)
1-20 (Least vulnerable)	1.05 (0.87-1.23)	1.04 (0.87-1.23)	1.06 (0.88-1.27)
Payer			
Commercial	1.01 (0.87-1.17)	0.98 (0.87-1.17)	1.07 (0.87-1.31)
Dual	0.95 (0.54-1.66)	0.91 (0.54-1.66)	1.15 (0.83-1.60)
Medicaid	0.9 (0.88-1.08)	0.98 (0.88-1.08)	0.98 (0.86-1.11)
Medicare Advantage	0.97 (0.91-1.04)	0.94 (0.91-1.04)	1.07 (0.97-1.18)
Medicare fee-for-service	1.02 (0.93-1.12)	1.04 (0.93-1.12)	0.95 (0.86-1.05)
Spending type			
Facility inpatient	1.03 (0.84-1.26)	1.02 (0.84-1.26)	1.06 (0.84-1.34)
Facility outpatient	1.03 (0.86-1.23)	1.03 (0.86-1.23)	1.04 (0.88-1.23)
Professional	1.01 (0.88-1.16)	0.98 (0.88-1.16)	1.11 (0.92-1.35)
Prescription drug	0.92 (0.81-1.05)	0.91 (0.81-1.05)	0.96 (0.81-1.13)
Ancillary	1.00 (0.71-1.43)	1.01 (0.71-1.43)	0.97 (0.83-1.14)

Abbreviations: RoRR, ratio of rate ratios; SVI, Social Vulnerability Index.

^a^ See the Methods section for details on how RoRRs are calculated. To evaluate telemedicine adoption’s overall impact, averaged over the effects within urban and rural subgroups, we also adjusted for an urbanicity main effect in addition to using an urbanicity and time interaction effect. In our sample, 1.8% of patients had unknown urbanicity.

Point estimates suggested that high-adopting areas had 4.4% (95% CI, −11.2% to 3.0%) fewer visits and 2.3% (95% CI, −18.9% to 17.8%) lower spending among urban populations, 2.5% (95% CI, −12.9% to 8.0%) lower spending for Medicaid-enrolled individuals, 5.3% (95% CI, −47.1% to 66.2%) lower spending for dual-eligible individuals, 3.0% (95% CI, −9.2% to 3.5%) lower spending for MA beneficiaries, and 1.5% (95% CI, −19.1% to 19.8%) lower spending among the most socially vulnerable populations. All subgroup analyses remained null.

Conversely, point estimates suggested that high-adopting areas had 3.4% (95% CI, −4.9% to 12.5%) greater visits and 3.8% (95% CI, −12.2% to 21.4%) higher spending among rural populations, 1.1% (95% CI, −12.84% to 17.25%) higher spending for commercially insured individuals, 1.0% (95% CI, −7.1% to 11.5%) higher spending for Medicare FFS beneficiaries, and 4.5% (95% CI, −12.7% to 23.1%) higher spending among the least socially vulnerable populations (Table 2). In sum, all these point estimates crossed the null and were not statistically significant.

The regional stratified results shown in eTable 6 in Supplement 1 revealed no significant differences in the association between telemedicine adoption and visits and spending based on region. The results from the sensitivity analyses are provided in the eResults in Supplement 1. Briefly, top line results were similar after redefining telemedicine exposure by 2022 levels of adoption (eTables 7-10 in Supplement 1). The negative control analysis using injury-related spending also showed null results similar to our main findings, with no significant differential changes between high and low telehealth adoption regions (RoRR, 1.09; 95% CI −0.82 to 1.44).

## Discussion

In this nationwide cohort analysis, we found that telemedicine adoption was not significantly associated with changes in utilization or spending. Point estimates suggested modest differences overall (2.4% fewer visits and 0.5% lower spending), but 95% CIs crossed the null. Importantly, this null finding was consistent across all examined subgroups, including urban and rural populations, all major payer types (commercial, Medicare FFS, MA, Medicaid, and dual-eligible), all levels of area-level social vulnerability, and all spending categories. These findings ease concerns about large spending increases resulting from nationwide telemedicine expansion. A negative control analysis using injury-related spending supported our identification strategy by showing no differential changes between high and low telehealth adoption regions. Our sensitivity analysis redefining telehealth exposure using 2022 adoption levels also produced similar results, further supporting the robustness of our findings. Taken together, these results suggest that continuing current telemedicine coverage is unlikely to meaningfully increase near-term spending—a timely and policy-relevant finding as predeductible telehealth coverage is now permitted in high-deductible health plans, and federal telemedicine flexibilities are set to expire on December 31, 2027, absent further congressional action.^15,16,51,52,53,54,55^

Our results are broadly consistent with recent quasi-experimental difference-in-differences analyses, which found that health system–level telemedicine adoption was associated with modest impacts on utilization and spending among Medicare FFS beneficiaries.^35^ Other influential work on Medicare FFS beneficiaries found that telemedicine was associated with modestly reduced 30-day postvisit spending, and reduced spending on low-value care, largely consistent with our findings.^17,37^ Beyond elucidating the multipayer and nationwide associations of telemedicine adoption across the entire PHE, our findings provide additional new knowledge, such as the heterogeneous association of telemedicine adoption by payer, geography, and social vulnerability.

Although point estimates suggested directional changes with reduced visits and spending overall, 95% CIs were wide and we cannot rule out smaller effects. Several factors may explain the imprecision of our estimates, including heterogeneity in telemedicine implementation across regions and payers, the ecological nature of our exposure measure, and the substantial disruption to health care utilization patterns during the pandemic period. Null findings across all subgroups, combined with our injury-related negative control analysis, provide reassurance that telemedicine expansion has not produced large spending increases or exacerbated disparities across these dimensions. Our findings are broadly concordant with several other studies showing a modest telemedicine-associated substitution effect of Medicare FFS visits and evaluation and management encounters, and MA encounters.^34,35,37,56^

Future research should supplement these analyses by examining the associations of telemedicine with a broad set of measures, including self-reported health status, patient experience, and other important health outcomes,^57,58,59^ and determine precisely in what settings, conditions, and modalities telemedicine can enhance patient-oriented outcomes equitably and at the lowest possible cost. For example, although telemedicine visits appear to offer quality of chronic disease care comparable to that of in-person visits,^60,61^ telemedicine may currently be less efficient in addressing acute conditions often requiring physical examinations, such as musculoskeletal complaints.^62^

### Limitations

This work has limitations. First, our analysis uses regional-level aggregated data, which precludes adjustment for individual patient characteristics beyond those captured in our baseline measures; accordingly, aggregated results may not apply to individual patients.

Second, the study reflects a large convenience sample of insured US individuals. Although this database has been shown to broadly reflect demographic characteristics of insured US individuals,^2,31,63^ these results may not generalize to the entire US or those lacking insurance.

Third, our study design precludes definitive causal inference in the context of nationwide telemedicine expansion without randomization. Additionally, unmeasured confounders such as digital literacy may affect both telehealth adoption and outcomes. Although our difference-in-differences design is robust to time-invariant differences between our exposure and comparison groups, it is potentially vulnerable to time-varying confounding. Our finding of approximately parallel prepandemic trends suggests that in the absence of the pandemic, the 2 groups would have evolved similarly, but it is possible that the pandemic itself may have caused trends to diverge independently of its differential impact on telemedicine adoption. Nevertheless, redefining telemedicine exposure in 2022 instead of 2020 and our negative control analyses yielded results consistent with our main findings, thereby lessening concerns about unobserved time-varying confounding factors in our study.^35^

Fourth, patients were assigned to HRRs according to 2024 zip codes, which may differ from their residence during the study period (2019-2023), potentially introducing selection bias if patients who moved differed systematically from those who did not. Fifth, claims databases lack comprehensive clinical context and behavioral metrics, and billing code errors may affect outcome measurement. Sixth, our study did not evaluate quality of care or health outcomes, and claims data did not permit reliable stratification by race or ethnicity, precluding examination of differential effects across racial and ethnic groups. Future work should examine whether telemedicine affects quality measures and health outcomes equitably across populations.

## Conclusions

Nationwide telemedicine adoption was not significantly associated with changes in visits or spending, either overall or when stratified by urbanicity, payer type, or area-level social vulnerability, easing concerns about large spending increases from telemedicine expansion. As policymakers weigh whether to extend Medicare telemedicine flexibilities beyond their 2027 expiration, our findings suggest limited near-term spending risk, although continued evaluation of longer-term effects on costs, health outcomes, and quality of care remains essential.

## Notes

Supplement 1. eMethods. eResults. eFigure 1. Cohort Tree of Primary Analysis Cohort eFigure 2. Comparison of Primary Analysis Cohort with American Community Survey, 2019 eFigure 3. Unadjusted Ambulatory Visit Rates and 2023 Inflation Adjusted Total Medical Spending Rates in Rural and Urban Areas, 2018-2023 eFigure 4. Per-Member-Per-Month (PMPM) Spending in 2019, by AHRQ Disease Category eTable 1. Sensitivity Analysis, Primary and Specialty Care Office Visit Rates Per Patient Per Year for Primary Analysis Cohort, 2019 eTable 2. Placebo Test for Parallel Trends by Social Vulnerability Index (SVI) (01/01/2018-12/31/2019) eTable 3. Placebo Test for Parallel Trends by Insurance Type (01/01/2018-12/31/2019) eTable 4. Placebo Test for Parallel Trends by Rurality (01/01/2018-12/31/2019) eTable 5. Placebo Test for Parallel Trends by Visit and Spending Subtypes (01/01/2018-12/31/2019) eTable 6. Telemedicine’s Association with Ambulatory Visits and Total Medical Spending Stratified by U.S. Census Region eTable 7. Telemedicine’s Association with Ambulatory Visits and Total Medical Spending Using 2022 HRR-Quintiles of Telemedicine Use as the Exposure by Payer and Social Vulnerability Index Quintile, and Rurality, 2019-2023 eTable 8. Telemedicine’s Association with the Utilization of Different Service Types Using 2022 Telemedicine Exposure eTable 9. Telemedicine’s Association with Total Medical Spending for Different Categories of Spending Using 2022 HRR-Quintiles of Telemedicine Use eTable 10. Average Monthly Counts of Patients by Demographic, 2018-2023 eReferences

Supplement 2. Data Sharing Statement
