# Supplementary material for: Telemedicine Adoption, US Ambulatory Visits, and Total Medical Spending, 2019-2023
Source: JAMA Netw Open. 2026 May 11;9(5):e2611835. doi: 10.1001/jamanetworkopen.2026.11835 (PMC13162073; doi:10.1001/jamanetworkopen.2026.11835)
Supplement: Supplement 1. — eMethods. eResults. eFigure 1. Cohort Tree of Primary Analysis Cohort eFigure 2. Comparison of Primary Analysis Cohort with American Community Survey, 2019 eFigure 3. Unadjusted Ambulatory Visit Rates and 2023 Inflation Adjusted Total Medical Spending Rates in Rural and Urban Areas, 2018-2023 eFigure 4. Per-Member-Per-Month (PMPM) Spending in 2019, by AHRQ Disease Category eTable 1. Sensitivity Analysis, Primary and Specialty Care Office Visit Rates Per Patient Per Year for Primary Analysis Cohort, 2019 eTable 2. Placebo Test for Parallel Trends by Social Vulnerability Index (SVI) (01/01/2018-12/31/2019) eTable 3. Placebo Test for Parallel Trends by Insurance Type (01/01/2018-12/31/2019) eTable 4. Placebo Test for Parallel Trends by Rurality (01/01/2018-12/31/2019) eTable 5. Placebo Test for Parallel Trends by Visit and Spending Subtypes (01/01/2018-12/31/2019) eTable 6. Telemedicine’s Association with Ambulatory Visits and Total Medical Spending Stratified by U.S. Census Region eTable 7. Telemedicine’s Association with Ambulatory Visits and Total Medical Spending Using 2022 HRR-Quintiles of Telemedicine Use as the Exposure by Payer and Social Vulnerability Index Quintile, and Rurality, 2019-2023 eTable 8. Telemedicine’s Association with the Utilization of Different Service Types Using 2022 Telemedicine Exposure eTable 9. Telemedicine’s Association with Total Medical Spending for Different Categories of Spending Using 2022 HRR-Quintiles of Telemedicine Use eTable 10. Average Monthly Counts of Patients by Demographic, 2018-2023 eReferences [file jamanetwopen-e2611835-s001.pdf]

Supplemental Online Content

Mafi JN, Vangala S, Cantor J, et al. Telemedicine adoption, US ambulatory visits, and total medical spending, 2019-2023. *JAMA Netw Open*. 2026;9(5):e2611835. doi:10.1001/jamanetworkopen.2026.11835

eMethods.
eResults.
eFigure 1. Cohort Tree of Primary Analysis Cohort
eFigure 2. Comparison of Primary Analysis Cohort with American Community Survey, 2019
eFigure 3. Unadjusted Ambulatory Visit Rates and 2023 Inflation Adjusted Total Medical Spending Rates in Rural and Urban Areas, 2018-2023
eFigure 4. Per-member-per-month (PMPM) spending in 2019, by AHRQ disease category
eTable 1. Sensitivity analysis, Primary and Specialty Care Office Visit Rates Per Patient Per Year for Primary Analysis Cohort, 2019
eTable 2. Placebo Test for Parallel Trends by Social Vulnerability Index (SVI) (01/01/2018-12/31/2019)
eTable 3. Placebo Test for Parallel Trends by Insurance Type (01/01/2018-12/31/2019)
eTable 4. Placebo Test for Parallel Trends by Rurality (01/01/2018-12/31/2019)
eTable 5. Placebo Test for Parallel Trends by Visit and Spending Subtypes (01/01/2018-12/31/2019)
eTable 6. Telemedicine's Association with Ambulatory Visits and Total Medical Spending Stratified by U.S. Census Region
eTable 7. Telemedicine's Association with Ambulatory Visits and Total Medical Spending Using 2022 HRR-Quintiles of Telemedicine Use as the Exposure by Payer and Social Vulnerability Index Quintile, and Rurality, 2019-2023
eTable 8. Telemedicine's Association with the Utilization of Different Service Types Using 2022 Telemedicine Exposure
eTable 9. Telemedicine's Association with Total Medical Spending for Different Categories of Spending Using 2022 HRR-Quintiles of Telemedicine Use
eTable 10. Average Monthly Counts of Patients by Demographic, 2018-2023
eReferences

This supplemental material has been provided by the authors to give readers additional information about their work.

eMethods

Data Source and Study Population

The data source for this study was the Milliman MedInsight Emerging Experience Database. MedInsight provides healthcare organizations with data warehousing, analytics, and benchmarks for 132 million patients (amounting to approximately 40% of the U.S. population or 44% of those with health insurance in 2019). Healthcare organizations include health insurance companies and health systems (networks of hospitals, physician practice groups, or both). MedInsight provides consulting services to these healthcare organizations, including data analysis, interpretation, and benchmarking. For each of these health care organizations, Milliman maintains a data use agreement (DUA); some of which allow for the sharing of de-identified claims data for research purposes, while others do not. Therefore, the MedInsight Research Database is a convenience sample of 75 health care organizations that agreed to provide de-identified claims data for research purposes from 2017-2023. MedInsight is not permitted to share any identifying information regarding these client health care organizations, even if they choose to volunteer de-identified data to the research database (eFigure 1).[1]

Our study's primary analysis cohort included US adults with age ≥18 years who were known to remain alive with continuous insurance coverage in one of the following insurance categories: Medicaid, Medicare-Medicaid (dual-eligible), Medicare fee-for-service, Medicare Advantage (MA), or commercial coverage between January 1, 2018 and October 31, 2023. Dual-eligible beneficiaries could be enrolled in either TM or MA. Note the cohort size of 3.04 million is much smaller than large number of patients in the

© 2026 Mafi JN et al. *JAMA Network Open*.

database, and this is mainly because very few patients have continuous and uninterrupted coverage over nearly a 5 year period. As noted in the manuscript, such a fixed longitudinal cohort was necessary to maximize causal inference by quasi-experimentally comparing outcomes among the same group of exposed and unexposed patients before vs after telemedicine expansion. This method also avoided confounding by shifts in the underlying population over time.

Telemedicine utilization was identified with current procedural terminology (CPT) codes for telemedicine visits. This includes telephonic visits: 99441, 99442, 99443, 98966, 98967, 98968; Virtual Check-In visits: G2012, G2010; Online E-visits: 99421, 99422, 99423, G2061, G2062, G2063, 98970, 98971, 98972; Remote Physiologic Monitoring visits: 99453, 99457, 99458; the following procedure modifiers: 95, G1, GT, G0; or the place of service (POS) codes 02 and 10.[1] Moreover, we did include some procedure codes that might be considered RPM: 99453 - Rem Mntr Physiol Param Setup; 99457 - Rem physiol mntr 1st 20 min; 99458 - Rem physiol mntr ea addl 20.

We examined several types of ambulatory care visits including primary care, specialty care, emergency department, and preventive screening visits. Multiple elements are used to assign visit types, such as the type of claim, POS code, provider specialty, diagnostic codes, procedure codes, and revenue codes. These visits were divided into primary care and specialty care mainly based on the Rendering Provider Specialty, where primary care included the categories: family medicine, general practice, general internal medicine, general pediatrics, and geriatrics. Primary care visits were defined as office visits with a primary care physician, preventive exam visits with a

© 2026 Mafi JN et al. *JAMA Network Open*.

primary care physician, or urgent care visits. Specialty care visits were defined as office visits with a specialist physician or preventive exam visits with a specialist physician. Emergency department (ED) visits were classified as any claim originating from an ED, regardless of whether the ED visit resulted in hospitalization. USPSTF/HRSA-recommended screening visits were defined as visits for breast cancer screening, colorectal cancer screening, contraceptive counseling, and HIV screening using the elements listed above.[1]

Spending was measured along 5 different categories: facility inpatient expenditures, facility outpatient expenditures, professional expenditures, prescription drug/pharmaceutical expenditures, and ancillary expenditures. Facility inpatient expenditures included, for example, hospitalizations. Facility outpatient expenditures included outpatient hospital department visits. Office visits were included in professional expenditures. Pharmaceutical expenditures involved the cost of filled prescriptions, while examples of ancillary expenditures were home health services and durable medical equipment.

This design was chosen to provide maximally conservative estimates of telemedicine's association with overall visit rates and spending rates before versus after the COVID-19 pandemic; therefore, we chose not to allow patients to enter/exit this longitudinal cohort over time. Despite improved internal validity, this fixed primary analytic cohort may be less representative of insured US adults, many of whom experience interruptions in coverage over time. Nevertheless, we have previously demonstrated that this database reflects similar demographic characteristics to U.S.

© 2026 Mafi JN et al. *JAMA Network Open*.

Census Bureau data, and we re-demonstrate this similarity with our study cohort in eFigure 2 below.[2]

Race and Ethnicity

Although we sought to examine the effects of telemedicine on racial and ethnic disparities in utilization of care, data contributors to both the monthly and annual research databases do not provide race and ethnicity data, especially for patients enrolled in Medicaid. Our primary analytic cohort was 17.4% white, 3.9% Black or African American, 0.44% Asian, 0.27% American Indian or Alaskan Native, 0.11% Native Hawaiian, and 1.8% some other race. 0.37% of patients had two or more races, and 75.8% of all patients had unknown race. Missing race and ethnicity data is a common issue with national claims data sets, particularly when considering Medicaid claims data.[3] The CDC's Social Vulnerability Index (SVI) measure does include racial and ethnic minority status at the U.S. Census tract level, but patient-level race and ethnicity data were not included in this analysis due to this large amount of missingness. Furthermore, due to strict DUAs, Milliman MedInsight's de-identified research database lacks surnames or residential address information typically used to create proxies for race/ethnicity in claims data, such as the RAND Bayesian Improved Surname Geocoding (BISG) method.

Proxies for socioeconomic vulnerability

We used the Social Vulnerability Index (SVI) a proxy for socioeconomic status to study telemedicine's effects on visits and spending. The SVI was designed by the Centers for Disease Control and Prevention (CDC) and the Agency for Toxic Substances and Disease Registry (ATSDR), and uses U.S. Census data to measure

© 2026 Mafi JN et al. *JAMA Network Open*.

social vulnerability at the census tract level.[4] Census tracts are subdivisions of counties. This SVI score is comprised of sixteen social factors organized into four SVI themes: Socioeconomic status (below 150% poverty, unemployed, housing cost burden, no high school diploma, no health insurance); Household characteristics (aged 65 or older, aged 17 or younger, civilian with a disability, single-parent households, English language proficiency); Racial and ethnic minority status (Hispanic or Latino (of any race); Black and African American; American Indian and Alaska Native; Asian; Native Hawaiian and Other Pacific Islander; Two or More Races; Other Races); Housing type & transportation (multi-unit structures, mobile homes, crowding, no vehicle, group quarters).[4] Each census tract receives a score for each of the four themes, as well as an overall SVI score. We used the overall score in our primary analysis. SVI was identified using the publicly available CDC/ATSDR Social Vulnerability Index data at the census tract level. These census tracts were then linked by 5-digit ZIP code to the MedInsight database using HUD-USPS ZIP Code Crosswalk data and guidelines published by the U.S. Department of Housing and Urban Development Office of Policy Development and Research (PD&R).[5]

Hospital Referral Regions

The Dartmouth Atlas of Health Care is an ongoing project to document variations in U.S. medical resource distribution using multiple data sources to discern information about national, regional, and local health care markets.[6] Hospital referral regions (HRRs) refer to geographic areas representing regional markets for tertiary medical care.[7] Broadly, HRRs are defined primarily by collections of zip codes with at least one hospital where hospitalized residents with Medicare had either a major cardiovascular

© 2026 Mafi JN et al. *JAMA Network Open*.

procedure or neurosurgery. They are secondarily assessed via population size and the proportion of all hospitalizations for area residents that occur in a hospital (or hospitals) inside the area. They are regionally adjusted to insure geographic contiguity. Patients were sorted into HRRs by cross-walking 5-digit zip code to HRR.

eResults

Sensitivity Analyses to Address Utilization Rates

To confirm that our baseline utilization rates were in line with established literature, we chose to compare the baseline primary and specialty care utilization rates of our 2019 Medicare fee-for-service insurance group to that of Barnett et. al, who examined primary care and specialty care visits for Medicare fee-for-service beneficiaries from 2009-2019.[8]

We compared primary and specialty care visit rates among Medicare fee-for-service beneficiaries in our primary analysis cohort in 2019 vs primary and specialty care visit rates among Medicare fee-for-service beneficiaries in Barnett et al.'s cohort in 2019. Barnett et al reported the mean annual number of primary and specialty care visits per beneficiary was 3.48 and 5.67 respectively, totaling 9.15 primary and specialty care visits per year, similar to our 8.6 primary and specialty care visits per-beneficiary-per-year (eTable 1) in our primary analysis cohort. We considered Barnett et al.'s rates to be comparable to our visit rates.

Sensitivity Analyses for Missing Data

We performed multiple sensitivity analyses to address missing data. First, we considered five types of missing data: (1) Missing patient demographic data was

© 2026 Mafi JN et al. *JAMA Network Open*.

generally low: 0.76% of patients in the primary analysis cohort had missing gender data, 3.9% had missing SVI data, 1.7% had unknown U.S. region data, and 1.8% had unknown rural status. Demographic data missingness did not differ by exposure to telemedicine; (2) missing data from organizations that did not contribute data to the MedInsight Database, which occurred infrequently (e.g., 9.1% of patients were excluded, eFigure 1); (3) missing data among patients with annual claims updates but lacking monthly claims updates, which upon evaluation previously revealed that demographics were similar to patients in our MedInsight research database[1]; (4) missing data via incomplete claims processing was rare with analyses revealing that 95.1-98.6% of claims were adjudicated by the time of this analysis.

To mitigate a contributor effect that resulted in missingness of pharmaceutical spending for Western Medicaid members in high-telemedicine-using HRRs from 2019 to 2021, we assumed that this subgroup's trends in non-pharmaceutical spending would also be reflected in pharmaceutical spending. Building upon this assumption, we utilized the overall share of total medical spending represented by pharmaceutical spending in this subgroup to impute the missing data. While pre-pandemic trends for pharmaceutical spending among Western Medicaid members were not parallel, placebo tests demonstrated pre-pandemic spending trends that fell within our pre-specified threshold for total medical spending and only marginally beyond our pre-specified threshold for significance for the pharmaceutical spending of Medicaid members across the entirety of the primary analysis cohort. Ultimately, results were unchanged with and without this imputation exercise (data not shown). We included the imputed data for comprehensiveness.

© 2026 Mafi JN et al. *JAMA Network Open*.

Additionally, all-cause hospitalizations were excluded in the visit-level difference-in-differences analyses a priori. This is because placebo trends testing of hospitalizations among those in higher vs lower HRR-quintiles of telemedicine-adoption in 2019 vs 2018 suggested non-pre-parallel trends (data not shown), similar to the behavioral health and substance use disorder visits noted in the manuscript. Nevertheless, hospitalizations were captured in the difference-in-differences analyses on spending, specifically via the inpatient spending subtype.

Finally, regarding the regression specification, it is: log(E[y]) = period + quintile + period * quintile + urban + urban * period + age + sex + comorbidities + offset(log(N)) (where y is either utilization or spending, period is pre- v. post-pandemic, quintile is top v. bottom telemedicine quintile, and n is the number of patients. The period*quintile term was used to estimate the RoRRs [after exponentiation]).

© 2026 Mafi JN et al. *JAMA Network Open*.

eFigure 1. Cohort Tree of Primary Analysis Cohort

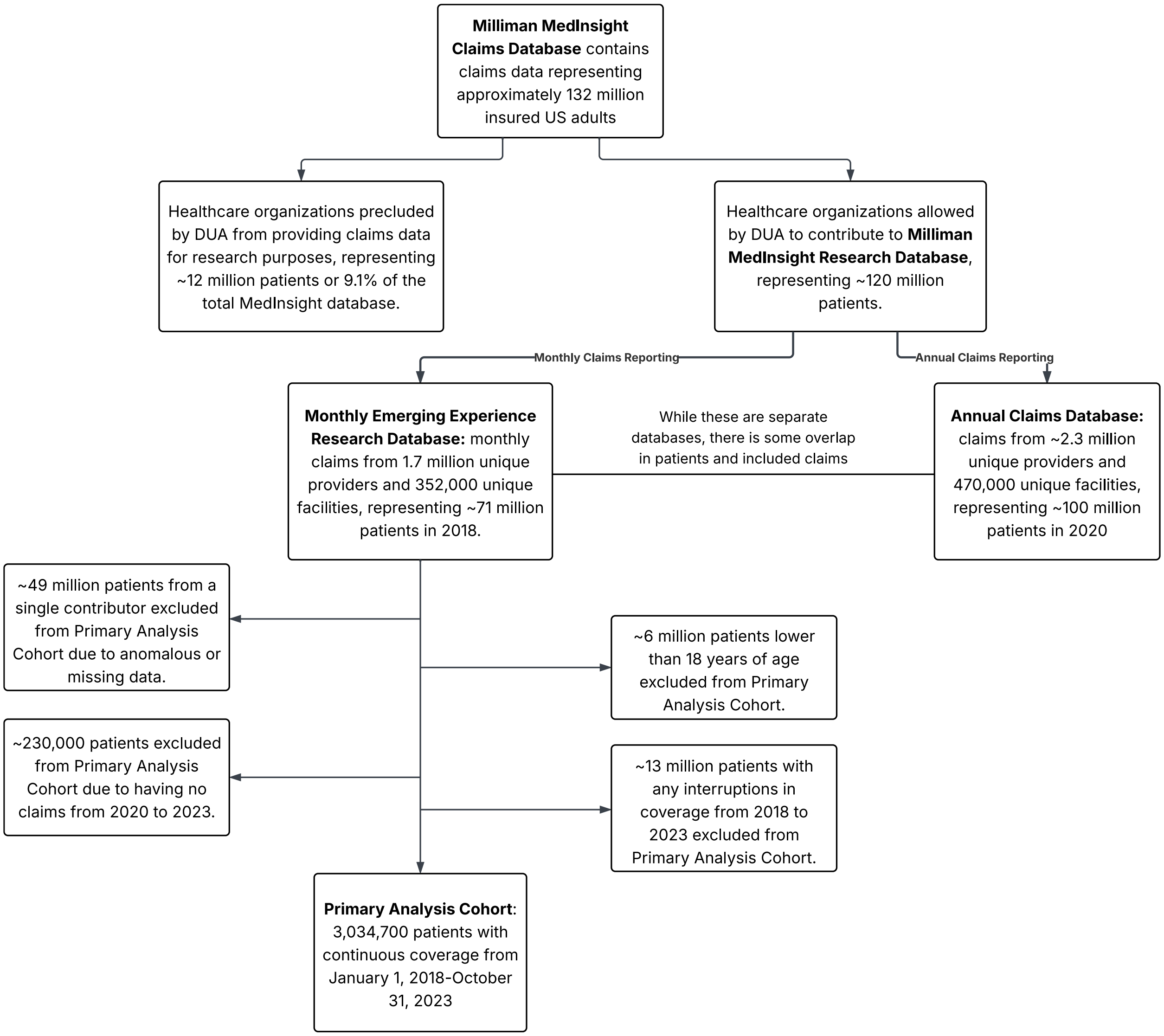

Publicly available data sources from the American Community Survey (ACS) estimated that there were 300 million US adults with health insurance in the United States in 2019. Using data from the NPPES (National Plan & Provider Enumeration System, source: https://nppes.cms.hhs.gov/), Milliman MedInsight provided us with the estimated number of providers by calculating a count of unique Type 1 (provider) National Provider Identifier (NPI) codes. Milliman MedInsight also provided us with the estimated number of facilities by calculating a count of unique Type 2 (facility) NPI codes. These estimates were rounded for accuracy because these databases are dynamic.

Note that Milliman's separate annually updated claims database (aside from negligible patient overlap) is excluded from the MedInsight research database because annual updates preclude examining contemporary care patterns during the pandemic.

Abbreviations: DUAs = data use agreements; NPPES = National Plan and Provider Enumeration System; Monthly Emerging Experience Database = claims data refreshed monthly to obtain all available claims data; Annual Claims Database = claims data updated once each year, representing health services that were rendered 18 months in the past.

© 2026 Mafi JN et al. *JAMA Network Open*.

eFigure 2. Comparison of Primary Analysis Cohort with American Community Survey, 2019

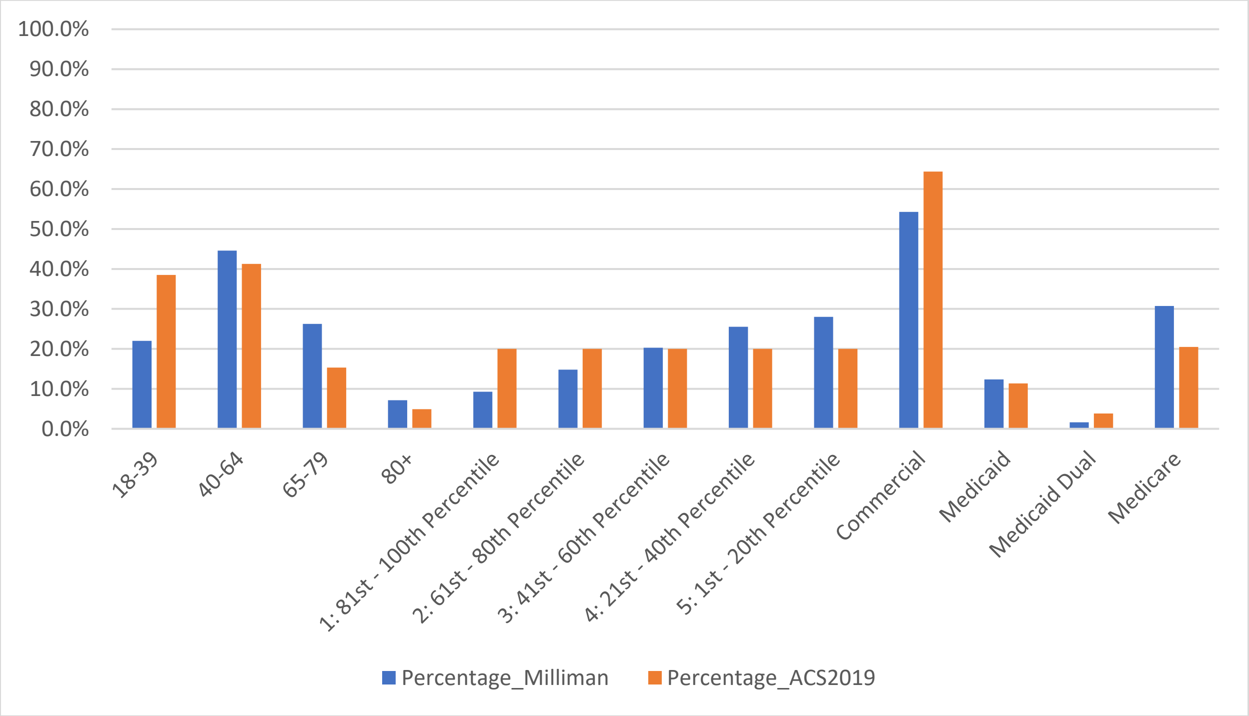

© 2026 Mafi JN et al. *JAMA Network Open*.

eFigure 3. Unadjusted Ambulatory Visit Rates and 2023 Inflation Adjusted Total Medical Spending Rates in Rural and Urban Areas, 2018-2023

eFigure 3A. Rural Ambulatory Visits per 100 Patients per Month

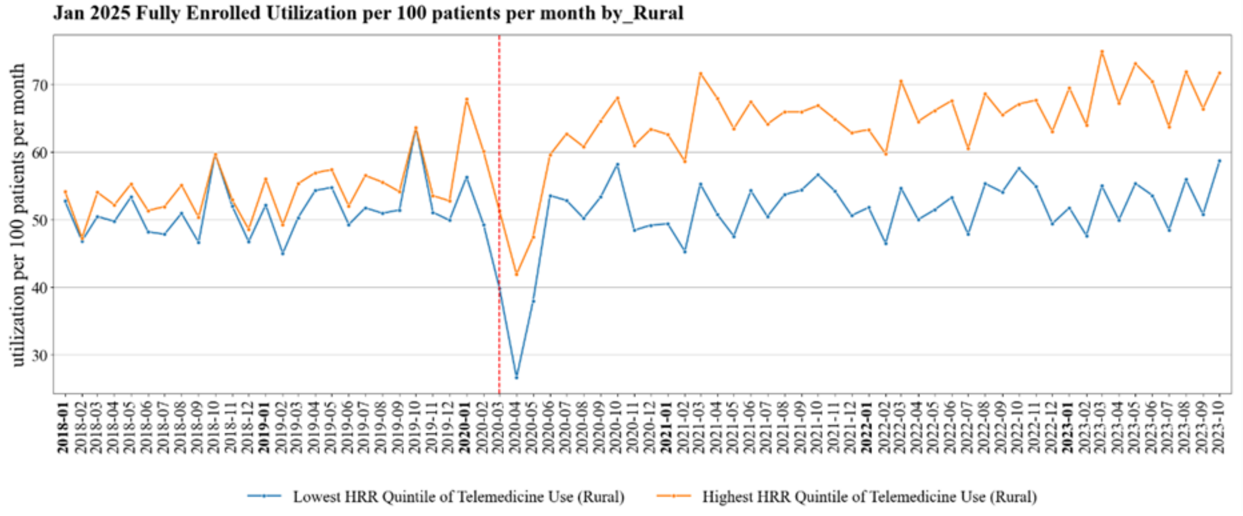

eFigure 3B Rural Per Member Per Month (PMPM) Total Medical Spending

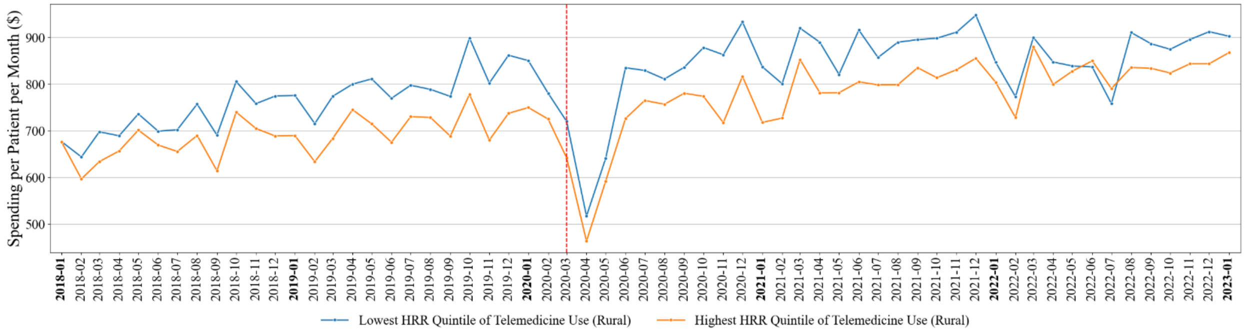

eFigure 3C. Urban Ambulatory Visits per 100 Patients per Month

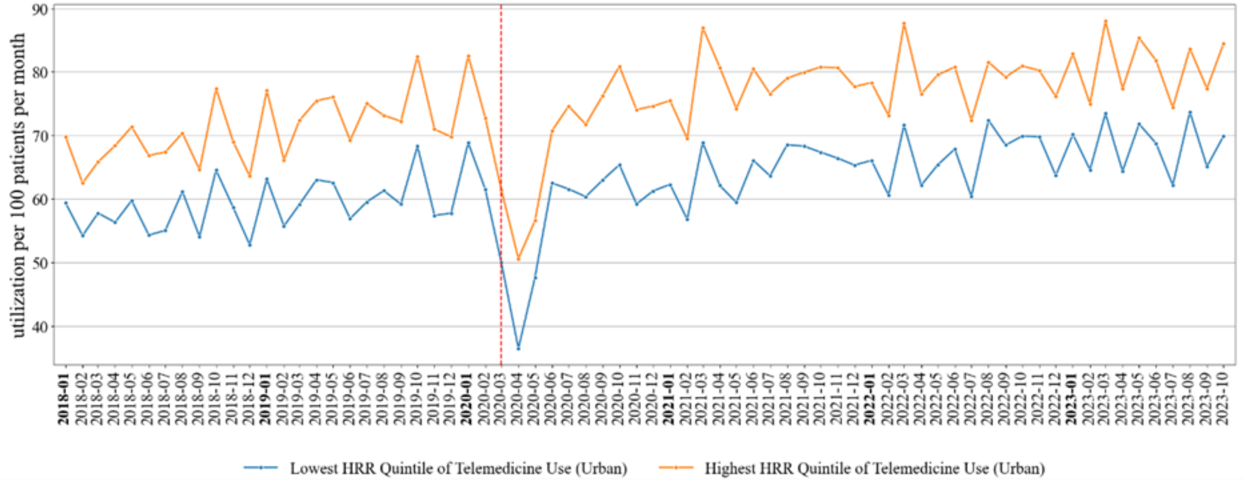

© 2026 Mafi JN et al. *JAMA Network Open*.

eFigure 3D. Urban Per Member Per Month (PMPM) Total Medical Spending

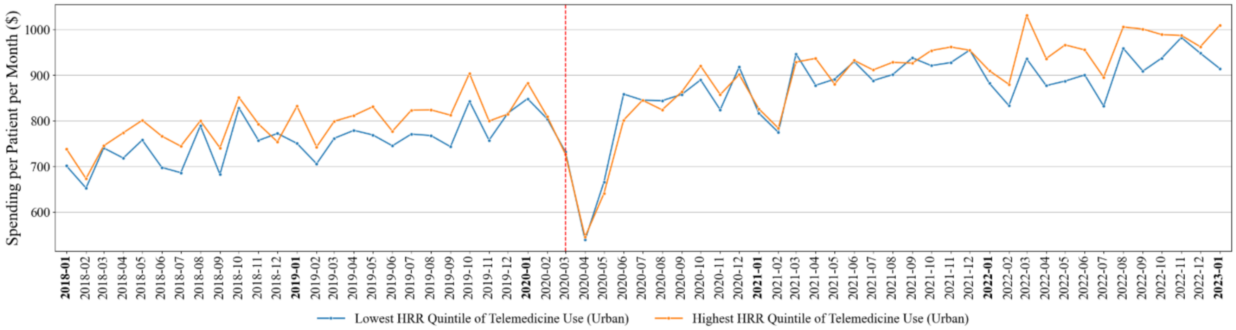

© 2026 Mafi JN et al. *JAMA Network Open*.

eFigure 4. Per-member-per-month (PMPM) spending in 2019, by AHRQ disease category

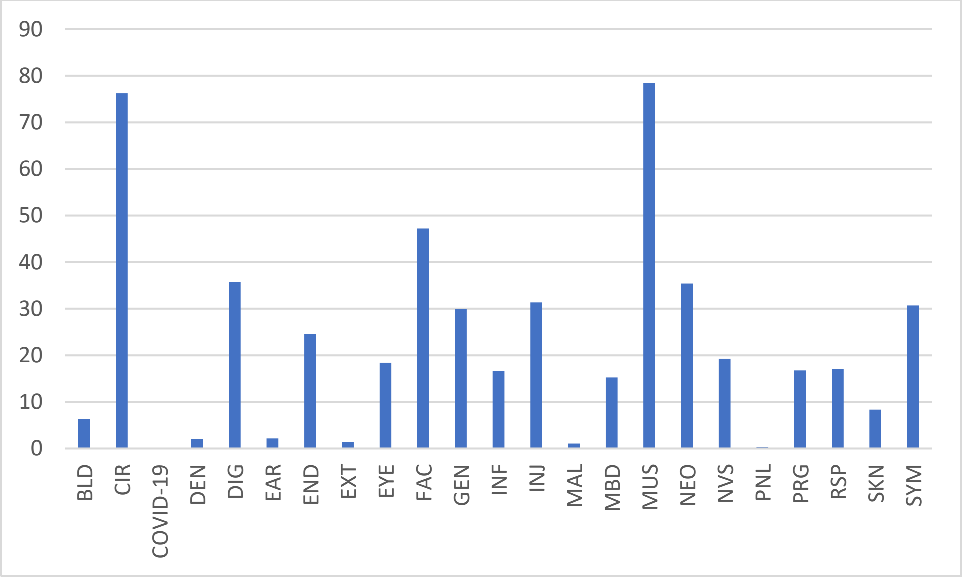

BLD: COVID-19; CIR: Diseases of blood and blood-forming organs, and certain disorders; DEN: Dental diseases and other diseases of the oral cavity; DIG: Endocrine, nutritional and metabolic system; END: Injury, poisoning and certain other metabolic diseases; EXT: Diseases of the eye and adnexa; EXT: Diseases of the digestive system and certain other contacts with health services; FAC: Factors influencing health status and contact with health services; INF: Diseases of the genitourinary system; GEO: Neoplasms; MUS: Mental, behavioral and neurodevelopmental disorders and diseases of the musculoskeletal system and connective tissue; PRG: Diseases of the skin and subcutaneous tissue; RSP: Diseases of the respiratory system and childbirth; SYM: Symptoms, signs and abnormal clinical laboratory findings, not elsewhere classified.

© 2026 Mafi JN et al. *JAMA Network Open*.

eTable 1. Sensitivity analysis, Primary and Specialty Care Office Visit Rates Per Patient Per Year for Primary Analysis, 2019

	Utilization rate per patient per year, 2019
**Primary Cohort**	
**Primary Care Visits (Primary Care Visits + Physicals)**	3.96
**Specialist Visits**	4.66
**Primary Care Visits + Specialist Visits**	8.62

© 2026 Mafi JN et al. *JAMA Network Open*.

eTable 2. Placebo Tests for Parallel Trends by Social Vulnerability Index (SVI) (01/01/2018-12/31/2019)

Service	Strata	Utilization	Spending
All	1: 81st - 100th Percentile	0.985 (0.931, 1.042)	0.992 (0.839, 1.172)
All	2: 61st - 80th Percentile	0.999 (0.956, 1.043)	1.008 (0.876, 1.160)
All	3: 41st - 60th Percentile	0.985 (0.955, 1.016)	0.996 (0.910, 1.090)
All	4: 21st - 40th Percentile	1.007 (0.959, 1.057)	1.023 (0.915, 1.144)
All	5: 1st - 20th Percentile	1.001 (0.959, 1.046)	1.050 (0.919, 1.198)
All	Unknown Overall SVI	1.017 (0.952, 1.086)	1.051 (0.861, 1.282)

For placebo pre-parallel trends testing, we used age-sex-diagnosis adjusted Poison regression models to calculate the relative rise in pre- vs post-pandemic visit rates among the exposure group vs the comparison group. This relative rise is reflected by the ratio of rate ratios (RoRR), derived from difference-in-differences estimated on the log-visit scale. These difference-in-differences analyses compared relative visit and spending rate changes during 2018 vs 2019 among those living in high- vs low-hospital referral region (HRR) quintiles of telemedicine use in 2020. An RoRR of 1 means that no change in the either visit rates or medical spending from 2018 to 2019 has occurred. RoRRs of less than 1 signify declines and may be interpreted as a percentage (e.g. an RoRR of 0.96 is interpreted as a 4% decrease relative to pre-pandemic rates). Conversely, RoRRs greater than 1 signify increases (e.g., an RoRR of 1.11 would be interpreted as an 11% increase). Each RoRR is accompanied by a 95% confidence interval. Overall, eTables 3-5 also show that stratifying by SVI, payer type, and rurality yield approximately parallel trends.[1]

© 2026 Mafi JN et al. *JAMA Network Open*.

eTable 3. Placebo Test for Parallel Trends by Insurance Type (01/01/2018-12/31/2019)

Service	Strata	Utilization	Spending
All	Commercial	1.005 (0.965, 1.047)	1.0029 (0.9376, 1.0727)
All	Dual	1.011 (0.777, 1.314)	0.9665 (0.5574, 1.6757)
All	Medicaid	1.021 (0.980, 1.064)	1.0696 (0.9493, 1.2052)
All	Medicare Advantage	0.970 (0.931, 1.010)	1.0366 (0.9780, 1.0987)
All	Medicare FFS	1.000 (0.965, 1.035)	1.0201 (0.9279, 1.1213)

See legend for eTable 2 for details and interpretation guidance.

© 2026 Mafi JN et al. *JAMA Network Open*.

eTable 4. Placebo Test for Parallel Trends by Rurality (01/01/2018-12/31/2019)

Service	Strata	Utilization	Spending
All	Rural	1.001 (0.960, 1.044)	0.9889 (0.867, 0.128)
All	Urban	0.997 (0.962, 1.034)	1.0295 (0.890, 1.191)

See legend for eTable 2 for details and interpretation guidance.

© 2026 Mafi JN et al. *JAMA Network Open*.

eTable 5. Placebo Test for Parallel Trends by Visit and Spending Subtype (01/01/2018-12/31/2019)

**Utilization**		
**Service**	**Urban Utilization**	**Rural Utilization**
Primary Care	0.988 (0.873, 1.118)	1.015 (0.973, 1.058)
Specialist Visit	1.000 (0.859, 1.164)	1.010 (0.919, 1.110)
ED Visit	0.989 (0.923, 1.061)	0.977 (0.908, 1.051)
Preventive Screening	1.021 (0.933, 1.117)	0.963 (0.844, 1.099)
**Spending**		
**Spending Type**	**Urban Spending**	**Rural Spending**
Facility Inpatient	1.0554 (0.8750, 1.2729)	0.9462 (0.7926, 1.1296)
Facility Outpatient	1.0454 (0.8490, 1.2874)	0.9833 (0.8510, 1.1362)
Professional	1.0502 (0.9023, 1.2225)	1.0446 (0.8727, 1.2503)
Pharmaceutical	0.9612 (0.8020, 1.1520)	0.9820 (0.8403, 1.1476)
Ancillary	1.0159 (0.6112, 1.6885)	0.9028 (0.7939, 1.0266)

See legend for eTable 2 for details and interpretation guidance.

© 2026 Mafi JN et al. *JAMA Network Open*.

eTable 6. Telemedicine's Association with Ambulatory Visits and Total Medical Spending Stratified by U.S. Census Region

	Strata	Visits Placebo	Visits	Spending Placebo	Spending
Census Region	Northeast	0.980 (0.946, 1.016)	1.005 (0.940, 1.075)	1.017 (0.914, 1.133)	0.999 (0.861, 1.158)
	Midwest	1.029 (0.946, 1.118)	1.064 (0.964, 1.174)	1.032 (0.854, 1.249)	1.077 (0.883, 1.312)
	South	0.974 (0.903, 1.050)	0.969 (0.883, 1.063)	0.974 (0.813, 1.180)	0.891 (0.723, 1.099)
	West	1.023 (0.915, 1.145)	1.054 (0.912, 1.219)	0.969 (0.754, 1.246)	1.215 (0.918, 1.608)

For our analyses stratified by census region, we used age-sex-diagnosis adjusted Poison regression models to calculate the relative rise in pre- vs post-pandemic visit rates among the exposure group vs the comparison group. This relative rise is reflected by the ratio of rate ratios (RoRR), derived from difference-in-differences estimated on the log-visit scale. These difference-in-differences analyses compared relative visit and spending rate changes during pre-(2019) vs post-telemedicine expansion (2021-2023) among those living in high- vs low-hospital referral region (HRR) quintiles of telemedicine use in 2020. An RoRR of 1 means that no change in the either visit rates or medical spending from pre-to-post pandemic-onset has occurred. RoRRs of less than 1 signify declines and may be interpreted as a percentage (e.g. an RoRR of 0.96 is interpreted as a 4% decrease relative to pre-pandemic rates). Conversely, RoRRs greater than 1 signify increases (e.g., an RoRR of 1.11 would be interpreted as an 11% increase). Each RoRR is accompanied by a 95% confidence interval. The placebo results of the table show that stratifying by region yield approximately parallel trends.[1]

© 2026 Mafi JN et al. *JAMA Network Open*.

eTable 7. Telemedicine's Association with Ambulatory Visits and Total Medical Spending Using 2022 HRR-Quintiles of Telemedicine Use as the Exposure by Payer and Social Vulnerability Index Quintile, and Rurality, 2019-2023

	Strata	Utilization Placebo	Utilization	Spending Placebo	Spending
Overall	All Patients	0.996 (0.965, 1.029	0.972 (0.909, 1.039)	0.992 (0.910, 1.0814)	0.983 (0.874, 1.106)
Coverage Type	Commercial	0.979 (0.936, 1.024	0.947 (0.849, 1.056)	0.994 (0.934, 1.058)	1.003 (0.867, 1.160)
	Dual	1.068 (0.934, 1.221	1.042 (0.914, 1.187)	0.933 (0.753, 1.155)	0.909 (0.732, 1.128)
	Medicaid	1.03 (0.979, 1.083	0.974 (0.898, 1.057)	1.051 (0.939, 1.176)	0.871 (0.760, 0.997)
	Medicare Advantage	1.001 (0.980, 1.022	0.992 (0.950, 1.036)	0.962 (0.902, 1.027)	1.017 (0.950, 1.089)
	Medicare FFS	1.013 (0.971, 1.057	1.021 (0.968, 1.077)	1.008 (0.933, 1.088)	0.984 (0.909, 1.065)
Social Vulnerability Index Quintile (higher percentile = more socially vulnerable)	1: 81st - 100th Percentile	0.986 (0.940, 1.034	0.949 (0.880, 1.023)	0.979 (0.881, 1.088)	0.956 (0.831, 1.099)
	2: 61st - 80th Percentile	0.997 (0.961, 1.034	0.929 (0.862, 1.001)	0.966 (0.880, 1.061)	0.937 (0.827, 1.061)
	3: 41st - 60th Percentile	0.986 (0.954, 1.019	0.990 (0.931, 1.052)	0.993 (0.902, 1.094)	0.979 (0.872, 1.099)
	4: 21st - 40th Percentile	1.000 (0.959, 1.043	0.972 (0.906, 1.042)	0.990 (0.908, 1.079)	1.002 (0.891, 1.126)
	5: 1st - 20th Percentile	1.003 (0.967, 1.040	0.986 (0.915, 1.062)	1.014 (0.923, 1.113)	0.998 (0.880, 1.132)
	Unknown Overall SVI	0.997 (0.886, 1.122	1.003 (0.888, 1.133)	0.857 (0.718, 1.022)	1.017 (0.803, 1.288)
Rurality	Rural	1.006 (1.000, 1.012)	1.064 (1.057, 1.071)	0.983 (0.983, 0.983)	0.995 (0.995, 0.995)
	Urban	0.994 (0.991, 0.997)	0.950 (0.947, 0.953)	1.005 (1.004, 1.005)	0.983 (0.983, 0.983)

See legend for eTable 6 for detail and guidance on interpretation. Note, in the manuscript, we emphasize the differences from our main results when the placebo RoRRs are close to 1.00.

© 2026 Mafi JN et al. *JAMA Network Open*.

eTable 8. Telemedicine's Association with Ambulatory Visits Using 2022 HRR-Quintiles of Telemedicine Use

Service	Strata	Utilization Placebo	Utilization Results
Primary Care	All Patients	0.996 (0.938, 1.05)	0.940 (0.871, 1.011)
Specialist	All Patients	0.997 (0.917, 1.09)	1.048 (0.954,1.145)
ED Visits	All Patients	0.979 (0.915, 1.08)	0.843 (0.765, 0.998)
Preventive Screening	All Patients	1.004 (0.921, 1.10)	0.959 (0.873, 1.092)

See legend for eTable 6 for detail and guidance on interpretation. The only difference is that telemedicine adoption is defined based on HRR-quintiles based on 2022 levels of telemedicine adoption.

© 2026 Mafi JN et al. *JAMA Network Open*.

eTable 9. Telemedicine's Association with Total Medical Spending for Different Categories of Spending Using 2022 HRR-Quintiles of Telemedicine Use

Service	Strata	Spending Placebo	Spending Results
Facility Inpatient	All Patients	1.0137 (0.8958, 1.1471)	0.9390 (0.781, 1.114)
Facility Outpatient	All Patients	0.9964 (0.8820, 1.1256)	1.0478 (0.859, 1.189)
Professional	All Patients	1.0162 (0.9519, 1.0849)	1.0214 (0.913, 1.118)
Pharmaceutical	All Patients	0.9373 (0.8249, 1.0650)	0.9144 (0.838, 0.065)
Ancillary	All Patients	0.9806 (0.7661, 1.2553)	0.9235 (0.726, 0.230)

See legend for eTable 6 for detail and guidance on interpretation. The only difference is that telemedicine adoption is defined based on HRR-quintiles based on 2022 levels of telemedicine adoption.

© 2026 Mafi JN et al. *JAMA Network Open*.

eTable 10. Average Monthly Counts of Patients by Demographic, 2018-2023

Age		Monthly Average
	18-39	690,302.45
	40-64	1,385,144.83
	65-79	768,033.00
	80+	210,724.72
Sex		
	Female	1,688,509.61
	Male	1,342,194.22
CCSR Count		
	0	70,233.77
	1	170,417.35
	2	193,314.87
	3	220,135.78
	4	241,478.61
	5+	1,946,137.99
Region		
	Midwest	1,276,059.54
	Northeast	625,990.83
	South	585,908.38
	West	514,942.26
Rurality		
	Rural	426,777.64
	Urban	2,572,932.36
Coverage		
	Commercial	1,666,540.12
	Medicaid	409,745.88
	Dual	32,412.00
	Medicare Advantage	508,739.00
	Medicare fee-for-servic	375,853.00
SVI		
	81st - 100th Percentile	297,065.86
	61st - 80th Percentile	441,119.93
	41st - 60th Percentile	544,361.86
	21st - 40th Percentile	759,628.35
	1st - 20th Percentile	894,008.13

Note: These demographic characteristics reflect the monthly average of patients with claims, and may differ from our demographic distributions at the unique patient level across the entire study time frame presented in the main results.

© 2026 Mafi JN et al. *JAMA Network Open*.

eREFERENCES

1. Mafi JN, Craff M, Vangala S, et al. Trends in US Ambulatory Care Patterns During the COVID-19 Pandemic, 2019-2021. *Jama*. Jan 18 2022;327(3):237-247. doi:10.1001/jama.2021.24294

2. Rockwell MS, Vangala S, Hadfield M, et al. Demographic Variation In US Outpatient Hydroxychloroquine And Ivermectin Use During The COVID-19 Pandemic. *Health Aff (Millwood)*. Feb 19 2025:101377hlthaff202400452. doi:10.1377/hlthaff.2024.00452

3. Nead KT, Hinkston CL, Wehner MR. Cautions When Using Race and Ethnicity in Administrative Claims Data Sets. *JAMA Health Forum*. Jul 1 2022;3(7):e221812. doi:10.1001/jamahealthforum.2022.1812

4. CDC/ATSDR Social Vulnerability Index (SVI) [Internet]. 2022 [cited 2023 Mar 17]; Available from: https://www.atsdr.cdc.gov/placeandhealth/svi/index.html.

5. HUD-USPS ZIP Code Crosswalk data and guidelines published by the United States Department of Housing and Urban Development Office of Policy Development and Research (PD&R). Accessed 03-20-2023, https://www.huduser.gov/portal/datasets/usps_crosswalk.html

6. Bronner KK, Goodman DC. The Dartmouth Atlas of Health Care - bringing health care analyses to health systems, policymakers, and the public. *Res Health Serv Reg*. Jul 27 2022;1(1):6. doi:10.1007/s43999-022-00006-2

7. Wennberg J, Cooper M. The Dartmouth Atlas of Health Care in the United States: The Center for the Evaluative Clinical Sciences [Internet]. American Hospital Publishing, Inc.; 1996:chap Part Nine, Strategies and Methods.

8. Barnett ML, Bitton A, Souza J, Landon BE. Trends in Outpatient Care for Medicare Beneficiaries and Implications for Primary Care, 2000 to 2019. *Ann Intern Med*. Dec 2021;174(12):1658-1665. doi:10.7326/m21-1523

© 2026 Mafi JN et al. *JAMA Network Open*.
