# Supplementary material for: Telemedicine Adoption, US Ambulatory Visits, and Total Medical Spending, 2019-2023
Source: JAMA Netw Open. 2026 May 11;9(5):e2611835. doi: 10.1001/jamanetworkopen.2026.11835 (PMC13162073; doi:10.1001/jamanetworkopen.2026.11835)
Supplement: Supplement 2. — Data Sharing Statement [file jamanetwopen-e2611835-s002.pdf]

Data Sharing Statement

Mafi. Telemedicine Adoption, US Ambulatory Visits, and Total Medical Spending, 2019-2023. *JAMA Netw Open*. Published May 11, 2026. doi:10.1001/jamanetworkopen.2026.11835

Data
Data available: No

Additional Information
Explanation for why data not available: The terms of our licensing agreement with Milliman MedInsight preclude us from being able to share the data freely.
